# The Influence of Light Rare-Earth Substitution on Electronic and Magnetic Properties of CoFe_2_O_4_ Nanoparticles

**DOI:** 10.3390/nano15151152

**Published:** 2025-07-25

**Authors:** Rareș Bortnic, Adam Szatmari, Tiberiu Dragoiu, Radu George Hategan, Roman Atanasov, Lucian Barbu-Tudoran, Coriolan Tiusan, Raluca Lucacel-Ciceo, Roxana Dudric, Romulus Tetean

**Affiliations:** 1 Faculty of Physics, “Babes Bolyai” University, Kogalniceanu 1, 400084 Cluj-Napoca, Romania; rares.bortnic@ubbcluj.ro (R.B.); adam.szatmari@ubbcluj.ro (A.S.); tiberiu.dragoiu@ubbcluj.ro (T.D.); radu.hategan@ubbcluj.ro (R.G.H.); atanasov.roman@ubbcluj.ro (R.A.); coriolan.tiusan@ubbcluj.ro (C.T.); raluca.lucacel@ubbcluj.ro (R.L.-C.); 2 Electron Microscopy Center “Prof. C. Craciun”, Faculty of Biology & Geology, “Babes-Bolyai” University, 5-7 Clinicilor St., 400006 Cluj-Napoca, Romania; lucian.barbu@itim-cj.ro; 3 Integrated Electron Microscopy Laboratory, National Institute for Research and Development of Isotopic and Molecular Technologies, 67-103 Donat St., 400293 Cluj-Napoca, Romania

**Keywords:** rare-earth cobalt ferrite nanoparticles, electronic structure, magnetic properties

## Abstract

Co_0.95_R_0.05_Fe_2_O_4_ nanoparticles with R = La, Pr, Nd, Sm, and Eu were synthesized via an environmentally friendly sol–gel method. The prepared samples were studied using X-ray diffraction measurements (XRD), transmission electron microscopy (TEM), X-ray photoelectron microscopy (XPS), and magnetic measurements. All compounds were found to be single phases adopting a cubic *Fd-3m* structure. EDS analysis confirmed the presence of Co, Fe, R, and oxygen in all cases. The XPS measurements reveal that the Co 2*p* core-level spectra are characteristic for Co^3+^ ions, as indicated by the 2*p*_3/2_ and 2*p*_1/2_ binding energies and spin–orbit splitting values. The analysis of the Fe 2*p* core-level spectra reveals the presence of both Fe^3+^ and Fe^2+^ ions in the investigated samples. The doped samples exhibit lower saturation magnetizations than the pristine sample. Very good agreement with the saturation magnetization values was obtained if we assumed that the light rare-earth ions occupy octahedral sites and their magnetic moments align parallel to those of the 3*d* transition metal ions. The ZFC-FC curves indicate that some nanoparticles remain superparamagnetic, while others exhibit ferrimagnetic ordering at room temperature, suggesting the presence of interparticle interactions. The M_r_/M_s_ ratio at room temperature reflects the dominance of magnetostatic interactions.

## 1. Introduction

Cobalt ferrite materials have garnered significant attention from scientists having in view both fundamental and applied research, owing to their excellent thermal stability, mechanical hardness, high coercivity, large magnetostriction coefficient, and notable magnetic anisotropy. These properties make them suitable for a wide range of applications, spanning from biomedical fields such as MRI contrast agents [1,2,3], DNA isolation [4,5,6], and magnetically controlled drug delivery systems [7,8,9,10] to electronics including magnetostrictive and gas sensors [11,12,13], optoelectronic devices, [14,15], microwave frequency components [16,17], and data storage media [18,19]. Given this broad application potential, numerous studies have been conducted by different research groups to investigate effects of rare-earth ion substitution on the properties of CoFe_2_O_4_ in various forms, such as bulk or nanostructured materials. For example, Vinosha et al. have demonstrated that CoZn ferrites possess a wide range of applications, including use in electronic devices, the biomedical field, the production of ferrofluids, and the development of radar-absorbing materials [20]. The magnetic and electrical properties of CoZn ferrites can be tailored through the doping of metals such as zinc, manganese, and magnesium, allowing the nanoparticles to be optimized for specific applications in both electrical and agricultural fields. In this context, the use of nanoparticles has had a notable impact during the COVID-19 pandemic. CoZn ferrites, in particular, have shown potential in the development of high-resolution tomography dyes for detecting viral strains within the body. Furthermore, CoZn ferrites may serve as promising nanomaterials for the design of nanocrystal-based therapeutics and medicines. Nanosized magnetic ferrite nanoparticles—such as CoFe_2_O_4_, MnFe_2_O_4_, ZnFe_2_O_4_, NiFe_2_O_4_, and CuFe_2_O_4_—have attracted significant attention over the past decade due to their unique properties and diverse applications in wastewater treatment. Among these, cobalt ferrite (CoFe_2_O_4_) stands out for its particularly promising performance in visible-light-driven photocatalysis, a rapidly advancing area of research. For a comprehensive overview, see the excellent review by Sukoviene et al. [21].

Rare-earth-ion-substituted spinel ferrites have emerged as promising materials for various advanced applications, including high-density magnetic recording, enhanced memory storage, magnetic fluids, and catalysis. Particular attention has been directed toward rare-earth-doped cobalt ferrites due to their potential in magneto-optical applications. This interest stems from their desirable properties such as high coercivity, strong anisotropy, high saturation magnetization, and high Curie temperature [22,23,24,25].

Substituting rare-earth elements (R) into CoFe_2_O_4_ has shown promise in tuning magnetic coupling, as evidenced by reductions in both the hyperfine field and Curie temperature—factors that contribute to enhanced sensitivity. Since magnetic performance is typically improved in well-crystallized materials at the nanoscale, rare-earth substitution becomes particularly valuable in this context. By reducing the ferrite particle sizes to the nanometer range, enhancement of potential magneto-optical or catalytic applications is expected by lowering noise and increasing sensitivity. For example, Hemasankari et al. reported improved photocatalytic activity in 5% Nd-doped CoFe_2_O_4_, achieving a degradation efficiency of 72% within 4 h—substantially higher than that of the undoped counterpart. These results suggest that doped CoFe_2_O_4_ nanoparticles hold promise for effective wastewater treatment applications [26]. Similarly, Oulhakem et al. investigated the photocatalytic performance of CoFe_2_O_4_ nanoparticles modified through low-level doping with Y^3+^ and Sm^3+^ cations [27]. Their findings demonstrated a significant enhancement in the degradation of Orange G dye, with photocatalytic efficiency increasing from 9.9% (undoped) to 64.63% for Sm^3+^-doped samples, 76.42% for Y^3+^-doped samples, and reaching 85.81% for co-doped samples after 60 min of UV–visible light exposure.

Rare-earth ions can introduce new magnetic interactions, thereby enhancing the magnetic properties of ferrites [28]. Previous studies have shown that doping with rare-earth elements can effectively suppress inhomogeneous magnetic spin structures [29]. This is attributed to the unique electronic configuration of R ions, which possess unpaired 4*f* electrons and exhibit strong spin–orbit coupling. Notably, the 4f electrons are well shielded by the outer 5*s*^2^ and 5*p*^6^ orbitals, making them relatively unaffected by the surrounding crystal field. When rare-earth ions are introduced into spinel ferrites, 4*f*–3*d* (4f-3d-5*d*) coupling occurs, playing a significant role in determining the magnetocrystalline anisotropy of the material [30,31,32].

The substitution of rare-earth (R) ions into the spinel lattice of cobalt ferrite has been extensively studied as a strategy to engineer its structural, magnetic, and optical properties. Owing to their larger ionic radii and anisotropic 4*f* magnetic moments, R ions significantly perturb the local crystal field and induce modifications in the cation distribution within the spinel framework. When R^3+^ ions replace Co^2+^ at octahedral sites, they can inhibit grain growth, reduce crystallite size, and generate lattice strain and symmetry distortions, leading to a deviation from the ideal cubic spinel structure [33,34,35,36,37]. These structural perturbations directly influence magnetic interactions, particularly through the modification of superexchange pathways. Moreover, the intrinsic magnetic behavior of R ions becomes increasingly dominant at cryogenic temperatures, offering functional advantages for low-temperature magnetic device applications. Systematic substitution with different R elements enables fine-tuning of the magnetic anisotropy, saturation magnetization, coercivity, and bandgap, thereby enhancing the applicability of R-doped cobalt ferrites in spintronic, magneto-optical, and high-frequency technological domains [38,39,40,41,42,43,44].

There are not many reports on rare-earth substitutions on Co sites; most of them are reporting the effect on the physical properties of substitutions on Fe sites. Dascalu et al. have reported the influence of Dy, Gd, and La substitutions on Fe sites in cobalt bulk ferrites [45]. A secondary phase with a perovskite-type structure was identified in concentrations ranging from 12% to 15%. The lattice parameter was found to be larger than that of stoichiometric cobalt ferrite, while the average crystallite size decreased; the saturation magnetization decreases while the coercive field increases with rare-earth substitution. These changes were attributed to the antiferromagnetic nature of the orthoferrite secondary phase at grain boundaries and to the higher atomic mass of R elements compared with that of iron. On the other hand, the La-doped cobalt ferrite exhibited a maximum magnetostriction coefficient that was higher than that of other ferrites. Daha et al. studied the effect of La, Ce, Gd, and Eu doping at Fe sites on cobalt ferrite properties [46]. They reported a decrease in the crystallite size due to the larger ionic radii of the rare-earth elements. The reduction in saturation magnetization was attributed to differences in the magnetic moments between the rare-earth (R) element and Fe, as well as to alterations in the structural properties of the samples. Additionally, the coercivity showed a slight increase with R doping, indicating that the incorporation of R elements influences not only the magnetization but also the magnetic anisotropy of the CoFe_2_O_4_ nanoparticles. The magnetic properties of the Co_0.5_Nd_0.5_Fe_2_O_4_ compound prepared by the co-precipitation method were reported by Mounkachi and co-workers [47]. According to the authors the saturation magnetization measured at room temperature decreased for doped sample while the coercive field increased from 1125.09 Oe to 2153.90 Oe. The *M*_r_/*M*_s_ ratio (*M*_r_ = remanent magnetization, *M*_s_ = saturation magnetization) was less than 0.5, a value characteristic of magnetostatic interactions between nanoparticles. The maximum energy product (BHmax) of the doped sample was 7% more than that of undoped cobalt ferrite.

Wu et al. studied the effect of R substitutions on the structural, magnetic, and adsorption properties of CoFe_1.9_R_0.1_O_4_, where R = Pr, Sm, Tb, Ho nanoparticles prepared by the hydrothermal method without any template and surfactant [48]. They confirmed the decreases in the lattice parameters and grain size for samples with R substitution. The saturation magnetizations for the doped sample decreases; this behavior is explained by the influence of local structure, cation distribution, and surface effect. The maximum adsorption capacities were found for samples with R = Sm or Ho, which had higher adsorption capacities than other oxide or mineral adsorbents, showing that they hold promise as excellent adsorbents for dye removal from wastewater. Pachpinde et al. prepared Pr_x_CoFe_2-x_O_4_ nanoparticles using the sol–gel method [49]. The sintered samples show presence of spinel cubic crystal structure and a secondary phase due to the presence of rare-earth Pr^3+^ ions. Pr^3+^ ions progressively occupy the B sites with increasing substitution levels. Co^2+^ ions show a clear preference for the B sites, while Fe^3+^ ions are distributed across both A and B sites throughout the entire compositional range. The system retains its ferrimagnetic ordering at room temperature for all studied samples. Introducing Pr^3+^ ions into the cobalt ferrite structure enhances both the saturation magnetization and coercive field; the higher coercivity compared to pure cobalt ferrite is attributed to increased magnetocrystalline anisotropy.

Zubair et al. [50] synthesized europium-doped cobalt ferrite nanoparticles, CoEu_x_Fe_2__−__x_O_4_, with x ≤ 0.12, using the co-precipitation method. Increasing Eu^3+^ doping concentrations led to a significant rise in coercivity, *H*c, between 944 and 9666 Oe and a corresponding decrease in saturation magnetization from 65 to 46 emu/g. This reduction in *M*_s_ was attributed to the substitution of magnetic Fe ions with non-magnetic Eu ions, weakening superexchange interactions. Additionally, the transition from a multi-domain to a single-domain structure contributed to the observed increase in *H*c. The study concluded that a controlled Eu^3+^ deficiency can enhance the magnetic properties of cobalt ferrite nanoparticles.

Previously we have shown that substitution with Zn or Mn at Co sites enhanced the magnetic properties of cobalt ferrite [51,52,53]. The obtained saturation magnetizations for compounds with Zn increased with the substitution of a few Co atoms compared with the pristine sample, while, in compounds with Mn substitution, the obtained *M*_s_ values were more than 50 percent larger than the previously reported values. The increased magnetic moments were explained by the preferential occupancy of Mn^4+^ ions on tetrahedral sites, while Mn^2+^ ions have the tendency to occupy octahedral sites. At the same time, the high quality and crystallinity of the prepared compounds—the nanoparticles are nearly monodomain—contributed to an improvement in the magnetic characteristics.

In this paper we report the electronic and magnetic properties of light rare-earth-doped cobalt ferrite. We characterized the prepared samples by X-ray diffraction measurements (XRD), transmission electron microscopy (TEM), scanning electron microscopy (SEM), X-ray photoelectron spectroscopy (XPS), and magnetic measurements. The XRD patterns confirmed that the compound are single phase without any impurities, a fact confirmed by EDS spectra too. The TEM images show nanoparticles with polygonal-to-spherical shapes. The nanoparticle dimensions determined from XRD and TEM are close, suggesting that the nanoparticles are almost monodomain with high crystallinity. The XPS measurements reveal that the Co 2*p* core-level spectra are characteristic for Co^3+^ ions, while both Fe^3+^ and Fe^2+^ ions are present in the investigated Co_0.95_R_0.05_Fe_2_O_4_ samples. XPS valence band spectra show contributions from the 3*d* states of Fe and Co, indicating that Co^3+^ ions are located in the octahedral sites. The magnetic properties of the studied samples are discussed based on XPS results. We chose to use light rare earths for substitution at the cobalt site having in view that there are only a few studies with R substitutions on Co sites and considering the large number of possible technical applications discussed above. We have shown, for the first time, the presence of Co^3+^ ions located preferentially on octahedral sites in cobalt ferrites. The computed magnetic moments using our cation distribution are almost the same as the experimental ones.

## 2. Materials and Methods

### 2.1. Sample Preparation

Cobalt ferrite nanoparticles partially substituted with 5 mol% trivalent rare-earth ions (La^3+^, Pr^3+^, Nd^3+^, Sm^3+^, and Eu^3+^) were synthesized via an environmentally friendly sol–gel method. The targeted composition was Co_0.95_RE_0.05_Fe_2_O_4_, where R represents the respective rare-earth dopant and replaces 5% of the Co^2+^ ions in the spinel lattice. To support this partial substitution, the synthesis pathway was designed to promote uniform cation dispersion and phase stability, while the expected local charge imbalance resulting from the introduction of R^3+^ in place of Co^2+^ is assumed to be compensated by structural adjustments within the spinel network, such as cation redistribution or oxygen vacancy formation.

The precursor salts used were iron(III) nitrate nonahydrate (Fe(NO_3_)_3_·9H_2_O), cobalt(II) nitrate hexahydrate (Co(NO_3_)_2_·6H_2_O), and the corresponding rare-earth nitrates—lanthanum(III) nitrate hexahydrate (La(NO_3_)_3_·6H_2_O), praseodymium(III) nitrate hexahydrate (Pr(NO_3_)_3_·6H_2_O), neodymium(III) nitrate hexahydrate (Nd(NO_3_)_3_·6H_2_O), samarium(III) nitrate hexahydrate (Sm(NO_3_)_3_·6H_2_O), and europium(III) nitrate hexahydrate (Eu(NO_3_)_3_·6H_2_O)—each with a purity of 99.9% and sourced from Alfa Aesar. Each synthesis was carried out based on a total yield of 2 mmol of final ferrite material.

Stoichiometric amounts of all metal nitrates were dissolved in Milli-Q water under vigorous magnetic stirring at 60 °C. After 1 h of homogenization, 14.9 mmol (~5 g) of sucrose was added as a natural chelating and polymerizing agent. The pH of the mixture was adjusted to approximately 2 using concentrated nitric acid (65% HNO_3_), after which 1 g of citrus-derived pectin was slowly incorporated under continuous stirring to prevent nanoparticle agglomeration and assist in gelation. The resulting solution was transferred to ceramic crucibles and heated in a sand bath at 240 °C for 24 h to evaporate water and promote gel formation. The obtained organic gel was then annealed in ambient air at 700 °C for 2 h to decompose the organic matrix and induce crystallization of the spinel phase.

The final product was a fine, well-crystallized nanopowder of composition Co_0.95_RE_0.05_Fe_2_O_4_, with R = La^3+^, Pr^3+^, Nd^3+^, Sm^3+^, or Eu^3+^. The method enables controlled substitution of Co^2+^ by RE^3+^ cations and provides a tunable platform for tailoring the magnetic, structural, and electronic properties of ferrite nanoparticles through a reproducible and sustainable synthesis approach.

### 2.2. Characterization

The crystal structure and crystallite sizes of Co_0.95_R_0.05_Fe_2_O_4_ nanoparticles were analyzed using X-ray diffraction (XRD) at room temperature with a Bruker D8 Advance diffractometer. Data were collected over a 2θ range of 20° to 80° in continuous mode, using a step size of 0.03° and a counting time of 5 s per step. Lattice parameters were determined through Rietveld refinement of the XRD patterns using the FullProf Suite software [37]. To account for instrumental broadening, the instrumental resolution function (IRF) was derived from a LaB_6_ NIST standard measured under identical experimental conditions. This IRF file was input into the refinement process to enhance the accuracy of structural analysis. The refinement employed Thompson–Cox–Hastings pseudo-Voigt functions for peak profile fitting. Parameters refined included the lattice constant, oxygen positional parameter, zero-shift correction, background coefficients, isotropic temperature factor, and peak-shape descriptors. The crystallite sizes of the prepared nanoparticles were estimated using the Debye–Scherrer equation:

$$D=\frac{k\lambda }{\beta \cos\theta }$$

where by *β* we denote the peak full width at half maximum (in radians) at the observed peak angle *θ*, by k the crystallite shape factor (considered 0.9), and by *λ* the X-ray wavelength.

The morphology of the studied samples was investigated by transmission electron microscopy (TEM) and scanning electron microscopy (SEM) using a Hitachi HD2700 CFEG STEM (Hitachi High-Tech, Tokyo, Japan) at 200 kV with secondary electron imaging capability. Energy-dispersive X-ray spectroscopy (EDS) measurements were also performed in order to check the composition of the prepared compounds.

X-ray photoelectron spectroscopy (XPS) spectra were collected at room temperature using a SPECS PHOIBOS 150 MCD system equipped with monochromatic Al Kα source (250 W, hν = 1486.6 eV), with hemispherical analyzer and multichannel detector. The pressure in the measuring chamber was around 10^−10^ mbar. Charge neutralization was used in all measurements. The binding energy (BE) of the obtained spectra was charge-referenced to the C 1s photoelectron peak at 284.6 eV. The high-resolution spectra were recorded using an analyzer pass energy of 30.

Magnetic measurements were performed in an external applied magnetic field up to 10 T in the temperature range 4.2–300 K, using a vibrating-sample magnetometer (VSM) from Cryogenic Limited London located at Ioan Ursu Institute.

## 3. Results

### 3.1. Morphology and Crystal Structure

**Figure 1:**
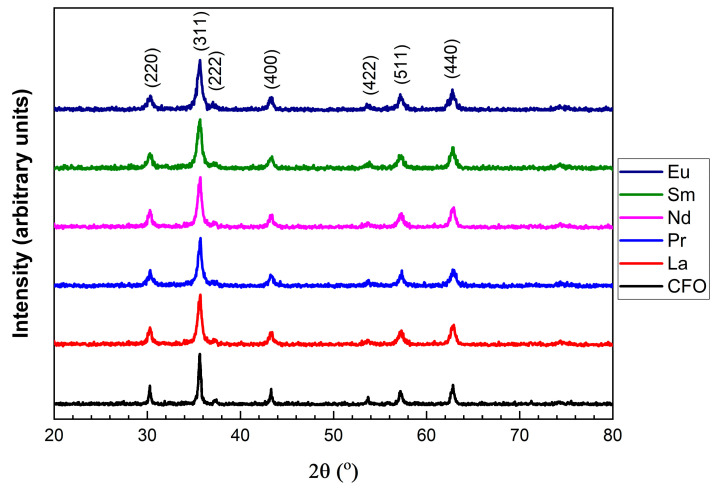
XRD patterns at room temperature of Co_0.95_R_0.05_Fe_2_O_4_ nanoparticles, where R = light rare-earth metals.

The X-ray diffraction (XRD) measurements, measured at room temperature, are presented in Figure 1, and all characteristic peaks for cobalt ferrites are present. The maximum intensity in the measured patterns was obtained for the (311) peak, which is commonly associated with the spinel ferrite structure. For comparison the XRD diffraction patterns of the pristine sample, CoFe_2_O_4_ (CFO), are also shown. The measured data confirmed that all prepared samples are single phases without any impurity phases and adopt a cubic *Fd-3m* structure.

**Figure 2:**
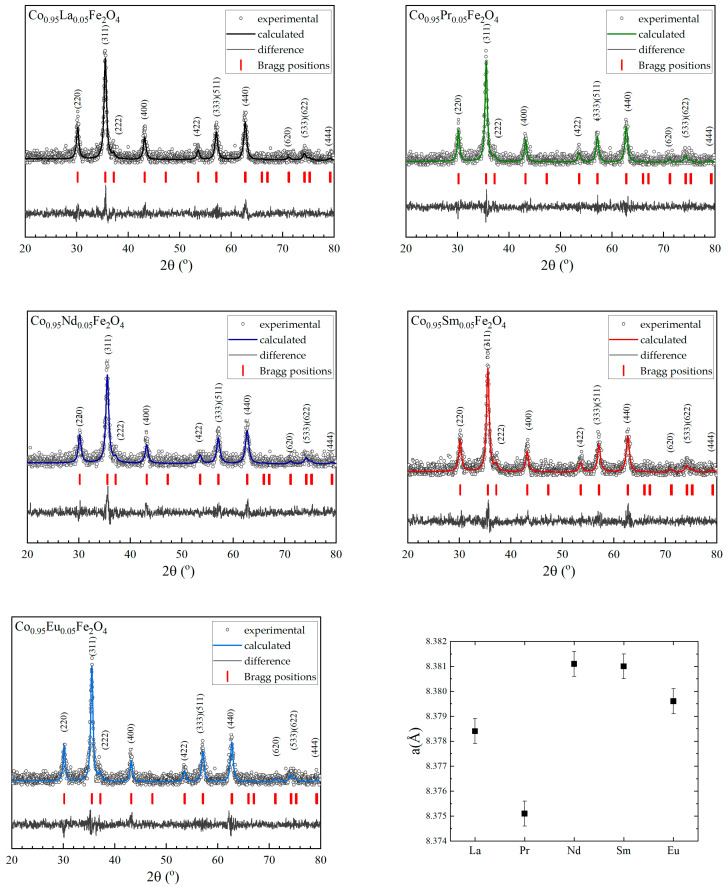
Rietveld refinement results for Co_0.95_R_0.05_Fe_2_O_4_ and R dependence of lattice parameters.

The theoretical patterns, the differences between them and the experimental ones, the Miller indices, and the rare earths’ dependence on the lattice parameters of the prepared compounds were calculated by Rietveld analysis using FullProf software [54] and are presented in Figure 2. The Rietveld analysis shows a very good match between theoretical and experimental curves, while, for all samples studied, the lattice parameter/unit cell volume have values around that of the pristine sample, having values between 8.375 nm and 8.381 nm. Having in view that the R^3+^ ionic radius is larger than that of Co ions, one can expect an increase in the lattice parameter. This small dependencies on R doping can be explained by the low concentration of light rare earths and are within the limit of experimental errors. The lattice parameter of cobalt ferrite was reported to be between 8.36 and 8.387 Å, and this value can change slightly depending on the synthesis method, temperature, and duration of heat treatment. The obtained values for the studied compounds are in the same range as those for CFO in previous reports. The crystallite sizes were calculated with the Debye–Scherrer formula, after subtracting the instrumental peak broadening; they have values between 19 nm and 21 nm for all investigated Co_0.95_R_0.05_Fe_2_O_4_ samples and are shown in Table 1.

**Figure 3:**
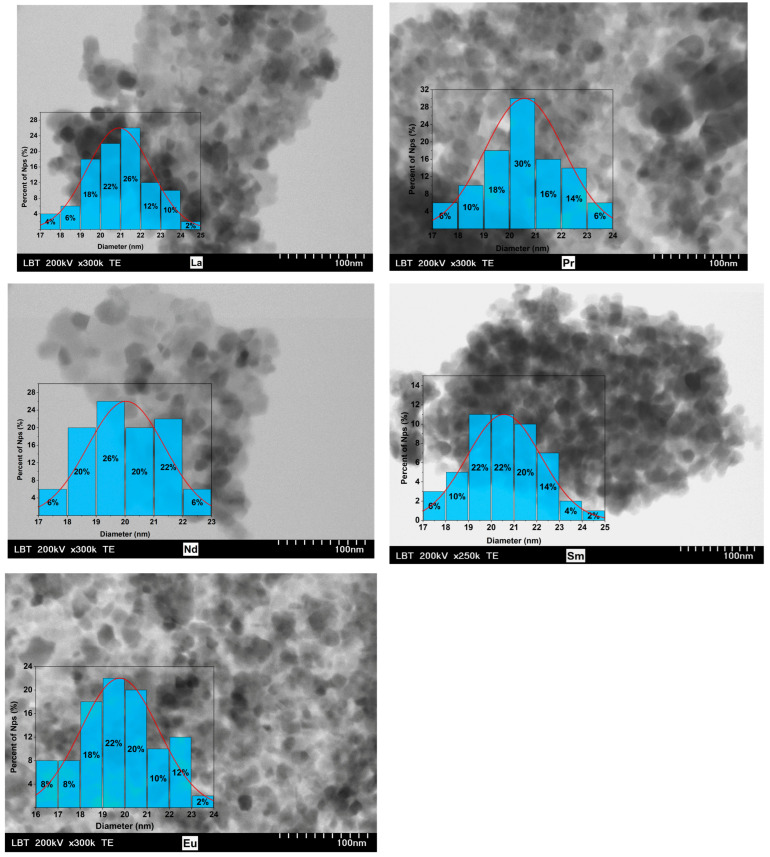
TEM images of Co_0.95_R_0.05_Fe_2_O_4_ nanoparticles. The distribution of the nanoparticles’ diameters is also shown.

Transmission electron microscopy (TEM) images for all studied compounds are shown in Figure 3. The image analysis reveals that well-defined nanoparticles are present, with polygonal-to-spherical shapes, which tend to agglomerate, probably due to the strong magnetic interactions that outweigh the relatively weak electrostatic repulsion. TEM images show nanoparticles ranging in size from 17 nm to 25 nm, with an average diameter closely matching the values derived from XRD measurements (Table 1).

This agreement suggests that most of the Co_0.95_R_0.05_Fe_2_O_4_ nanoparticles are monodomain and exhibit high crystallinity. However, the nanoparticles are notably polydisperse, and the error bars associated with the TEM measurements indicate that the average particle sizes may be slightly larger than those estimated from XRD data.

**Figure 4:**
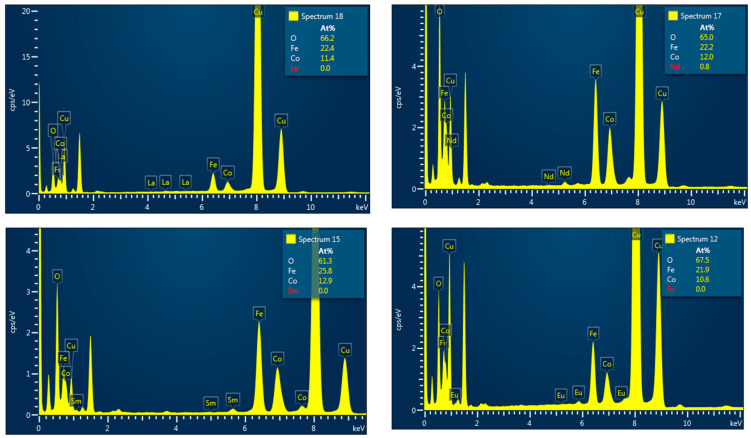
EDS spectra for the compounds with R = La, Nd, Sm, and Eu.

Energy-dispersive X-ray analysis (EDS), used in conjunction with scanning electron microscopy (SEM), enables identification and quantification of elements on or near the surface of nanomaterials to generate elemental maps. However, as X-rays are generated from a region approximately 2 µm deep, EDS lacks precision for surface-specific characterization. EDX can detect the composition and quantity of heavy metal ions near or at the sample surface, though it is less effective for elements with atomic numbers below 11. Advanced X-ray techniques such as X-ray photoelectron spectroscopy (XPS) could be further used for nanomaterial characterization. Elemental analysis performed by EDS measurements has confirmed the presence of Co, Fe, R, and O elements in all studied compounds. The EDS spectra for Co_0.95_R_0.05_Fe_2_O_4_ of the samples with R = La, Nd, Sm, and Eu are given in Figure 4 (the Cu grid lines are also shown). The EDS spectra confirm the absence of any impurities in the nanoparticles. The atomic concentrations presented in the right-hand frame of the spectra showed zero concentrations of light rare earths even though their characteristic lines are present in the spectra. Energy-dispersive X-ray spectroscopy generally provides good accuracy for major elements, with a typical relative uncertainty of ±2% to ±5%. However, accuracy tends to decrease for minor or trace elements, as well as for certain sample types. Probably, the small amount of doping compared with other elements is within the limit of the calculated errors.

**Figure 5:**
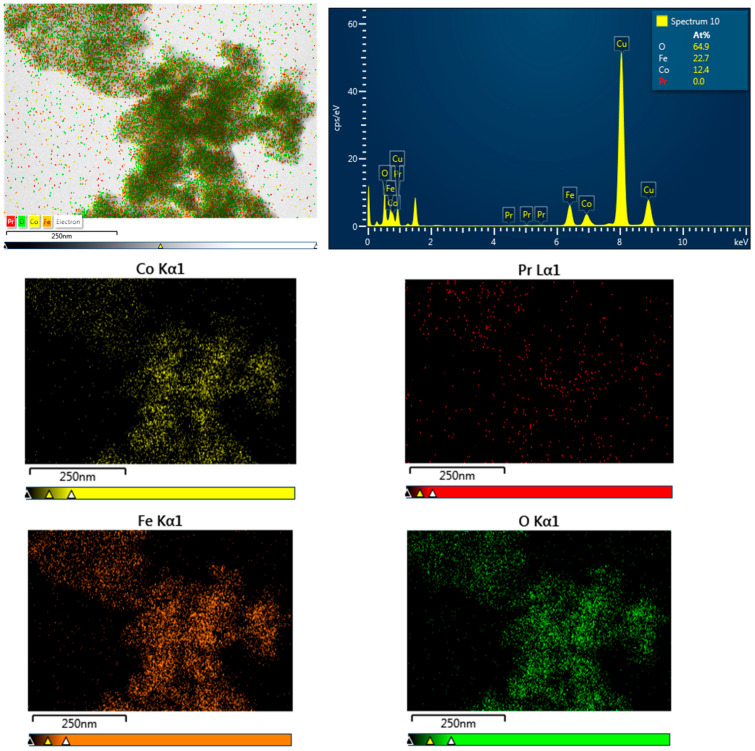
EDS spectra and the elemental mapping of Co_0.95_R_0.05_Fe_2_O_4_ nanoparticles.

Figure 5 presents the EDS spectra and the elemental mapping for the sample with R = Pr, revealing that Co, Pr, Fe, and O are distributed nearly homogeneously throughout the selected area. Comparable distributions were observed in all the samples analyzed. The elemental compositions of the studied samples are close to the expected stoichiometric ratios.

### 3.2. XPS Results

XPS analysis of Fe 2*p* and Co 2*p* core levels can provide valuable information about the ionic states in oxides. Although the transition metals’ 2*p* peaks are quite broad due to unresolved multiplet splitting, the position of the main 2*p*_3/2_ and 2*p*_1/2_ lines and that of the satellites resulting from final state effects can be used to identify the oxidation state of the ions [55,56,57,58,59,60].

**Figure 6:**
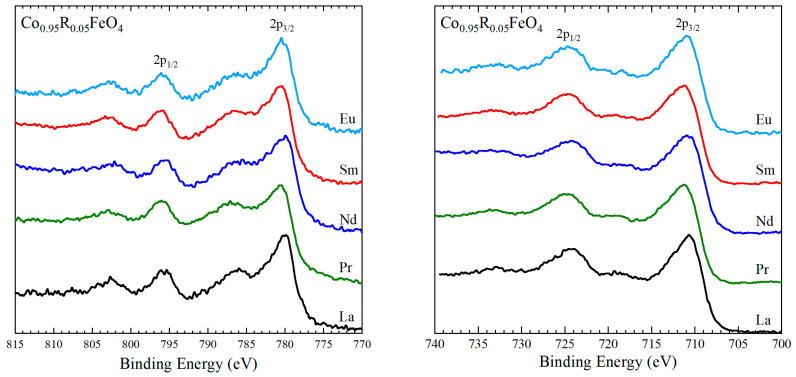
XPS Co 2*p* core-level spectra (**left**) and Fe 2*p* core-level spectra (**right**).

The recorded XPS spectra in the Co 2*p* region are presented in Figure 6 (left). They consist of two main signals, corresponding to the 2*p*_3/2_ and 2*p*_1/2_ states, and two satellites located at 6–7 eV higher energies than the main signals. The energies corresponding to the centers of the main peaks, listed in Table 2, are close to the values reported for Co^3+^ ions in CoFe_2_O_4_ [59,61]. The spin–orbit splitting of about 15.3–15.4 eV is close to the value of 15 eV found for cobalt(III) compounds [58], confirming that Co is predominantly present as Co^3+^ in the investigated Co_0.95_R_0.05_Fe_2_O_4_ nanoparticles.

The XPS spectra of the Fe 2*p* core level recorded for Co_0.95_R_0.05_Fe_2_O_4_ nanoparticles are shown in Figure 6 (right). The main 2*p*_3/2_ and 2*p*_1/2_ lines are centered at about 711 eV and 724.4 eV in all samples. Typical 2*p*_3/2_ binding energies in spinels are around 709 eV for Fe^2+^ ions in Fe_2_SiO_4_ [57] and about 711.9 eV for Fe^3+^ ions CoFe_2_O_4_ [60]. The presence of the satellite structures at 8 eV higher binding energies than the main lines is also proof of the presence of Fe^3+^ [55]. The large width of the 2*p*_3/2_ and 2*p*_1/2_ peaks and their asymmetric shape suggest a complex structure of the Fe 2*p* XPS spectra as does the presence of satellites closer to the main signals, originated from Fe^2+^ ions [57]. Hence the XPS spectra indicate the presence of both Fe^2+^ and Fe^3+^. A precise Fe^3+^/Fe^2+^ ratio is difficult to assess due to the overlap with the Co LMM Auger signal centered at 713 eV.

**Figure 7:**
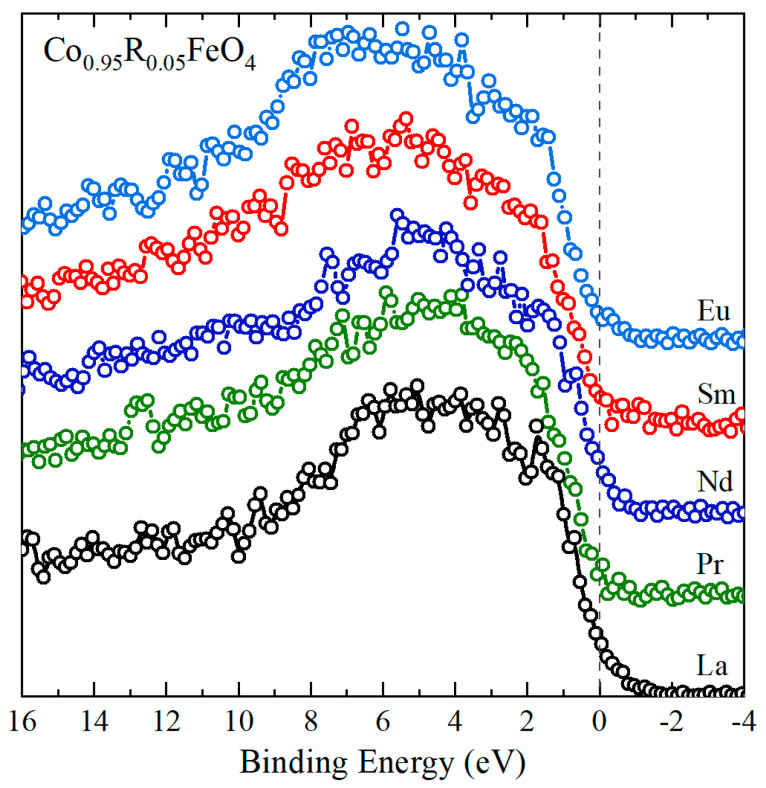
XPS spectra in the valence band region for Co_0.95_R_0.05_Fe_2_O_4_ nanoparticles.

The valence band spectra (Figure 7) arise from the specific contributions of the Co, Fe, O, and rare-earth ions. Below 3 eV the main contributions in the XPS spectra are from Co 3*d* and Fe 3*d* states [62,63,64]. According to Ran et al. [62], in Co-doped Fe_3_O_4_, the spectral signal below 0.5 eV originates from 3*d* bands of Fe^2+^ ions, while, in the 0.5–3 eV region, the XPS signal is assigned to the 3*d* states originating from Fe^3+^ and Co^3+^ ions. Similar assignments were reported for spinel cobaltite oxide thin films, with the valence band spectrum of ZnCo_2_O_4_ being assigned to the six-electron-occupied 3*d t_2g_* Co states with some hybridization with O 2*p* [64]. The feature at about 1.6 eV, noticeable especially in the La-doped sample, is attributed to 3*d*-derived states from Co^3+^ in the octahedral sites, as suggested by the valence band photoemission studies on Co_3_O_4_ films [63,65,66]. At binding energies larger than 3 eV, the O 2*p*-derived emissions are the most intense, while, above 7 eV, the 4*f* contributions from the rare-earth ions become visible, especially in Co_0.95_Sm_0.05_Fe_2_O_4_ and Co_0.95_Eu_0.05_Fe_2_O_4_. The density of states near the Fermi level is low in all samples, with no significant changes related to the rare-earth doping, suggesting that the rare-earth substitution has very little influence on the amount of Fe^2+^ in the doped cobalt ferrites.

### 3.3. Magnetic Measurements

**Figure 8:**
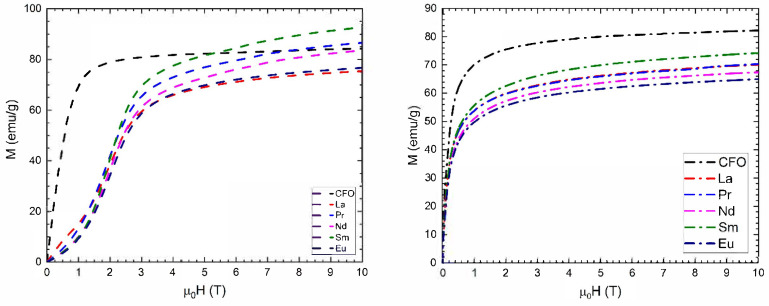
Magnetization isotherms of Co_0.95_R_0.05_Fe_2_O_4_ nanoparticles measured at 4.2 K (**left**) and 300 K (**right**). CFO isotherms are also shown.

The temperature variations of saturation magnetizations measured at 4.2 K and 300 K under an external magnetic field up to 10 T are presented in Figure 8. In all studied samples, saturation was not attained in fields up to 10 T. At 4.2 K, the magnetization curve displays inflection points around an applied magnetic field of 1.7 T, which can be attributed to the presence of a small fraction of superparamagnetic nanoparticles, along with the predominant ferrimagnetic ordering in the sample.

The saturation magnetizations, *M*_s_, at 4.2 K and 300 K of Co_0.95_Zn_0.05_Fe_2_O_4_ nanoparticles were calculated from magnetization isotherm measurements using the approach-to-saturation law:

$$M=M_{s}\left(1-\frac{a}{H}\right)+\chi_{0}H$$

where by *a* we have denoted the coefficient of magnetic hardness and by $\chi_{0},\;$a Pauli-type contribution. These values are lower for the doped samples compared with the pristine one, though they are generally higher or comparable with those reported in previous studies, which were between 66.8 emu/g and 73.4 emu/g. For example, values of 55 emu/g were reported for the CoEu_0.06_Fe_1.94_O_4_ sample [50], 59 emu/g for Pr_x_CoFe_2-x_O_4_, [49], 48.2 emu/g in the case of Pr_0.1_CoFe_1.9_O_4_ [46], 56.5 emu/g for La_0.1_CoFe_1.9_O_4,_ [46], and 60 emu/g for CoFe_1.9_R_0.1_O_4_ with R = Pr or Sm [48]. Prior research has indicated that rare-earth doping in nanocrystalline cobalt ferrite can influence magnetic properties, primarily due to changes in cation distribution between the tetrahedral and octahedral sites [67,68,69,70,71]. The magnetization values measured at 300 K under a 10 T magnetic field exhibit a trend like that of the saturation magnetization observed at 4.2 K.

The total magnetic moments calculated having in view our proposed cation distribution closely match the experimental values observed at 4.2 K (see Table 3). The magnetic moments per ion were assumed as follows: 3 μ_B_ for Co^2+^, 4 μ_B_ for Fe^2+^, 5 μ_B_ for Fe^3+^, 0 μ_B_ for La^3+^ and Eu^3+^, 3.2 μ_B_ for Pr^3+^, 3.27 μ_B_ for Nd^3+^, and 0.71 μ_B_ for Sm^3+^. Within each sublattice, the magnetic moments of ions in octahedral and tetrahedral sites are aligned parallel to one another, while the two sublattices are aligned antiparallelly. Additionally, the magnetic moments of light rare-earth ions are parallelly oriented to those of the 3*d* transition metals. In our calculations, we assumed that R moments are situated at octahedral sites and are parallel to the Co and Fe moments at the same sites, consistent with their behavior as light rare-earth elements. For calculation we impose that the total metallic ion valence is 8^+^. The best agreement with experimental data, considering the constraints, was achieved when most cobalt ions were in the 3^+^ valence state, as suggested by XPS measurements.

**Figure 9:**
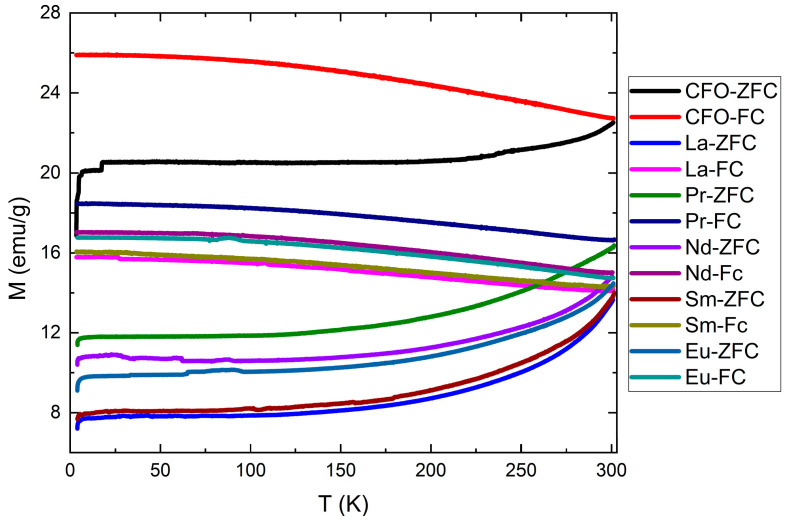
The temperature dependencies of ZFC and FC curves.

A deeper understanding of the magnetic behavior of ferrite magnetic nanoparticles (MNPs) can be achieved by analyzing the temperature-dependent magnetization using two standard measurement protocols: zero field cooling (ZFC) and field cooling (FC). A notable feature of the ZFC magnetization curve is the presence of a peak that marks the average transition from the ferromagnetic to the superparamagnetic state. This peak corresponds to the blocking temperature (*T*_B_). Additionally, the temperature at which the ZFC and FC curves begin to diverge—referred to as the irreversibility temperature—is generally higher than *T*_B_. In the case of monodisperse and non-interacting single-domain nanoparticles exhibiting superparamagnetic behavior, the blocking temperature is the same as the irreversibility temperature. In our samples, however, the ZFC magnetization peak emerges near room temperature and extends to higher temperatures as can be seen in Figure 9. The ZFC and FC curves converge around or slightly above room temperature. While the FC magnetization remains nearly constant with a small increasing tendency, the ZFC magnetization decreases with decreasing temperature. This behavior suggests that some nanoparticles remain superparamagnetic, whereas others become ferrimagnetically ordered at room temperature, indicating the presence of interparticle interactions.

**Figure 10:**
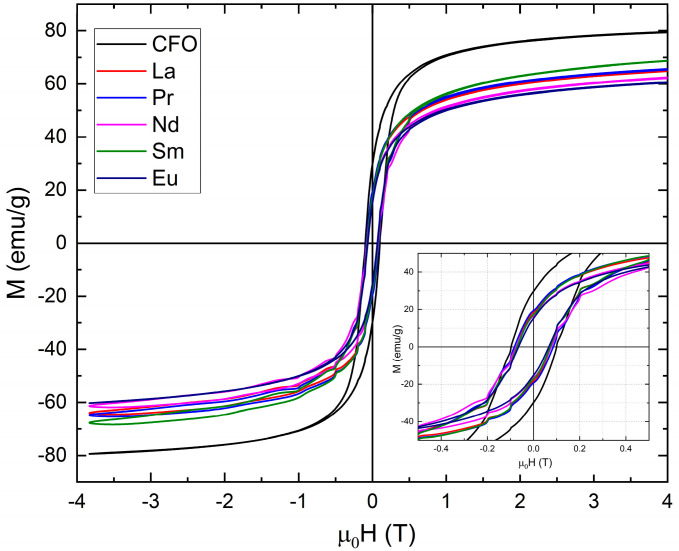
Hysteresis loops recorded at room temperature for Co_0.95_R_0.05_Fe_2_O_4_ nanoparticles.

The magnetic behavior of Co_0.95_R_0.05_Fe_2_O_4_ nanoparticles at room temperature was also examined by measuring hysteresis loops in the range of −4 T to 4 T and is shown in Figure 10 with a zoom-in in the picture frame. The coercive field has values around 0.06–0.07 T for all studied compounds. The coercive fields and remanent magnetization values are shown in Table 4.

Starting from the coercive field values and the saturation magnetizations, we calculated the anisotropy constant (K) at 300 K using the following relation:

$$K≅\mu_{0}H_{C}M_{S}$$

where *μ*_0_ is the vacuum magnetic permeability; *H*_c_ is the coercive field; and *M*_s_ is the saturation magnetization. Further, we calculated the blocking temperature, *T*_B_, using crystalline sizes determined by the XRD and TEM measurements, using the following formula:

$$T_{B}=\frac{K\;V}{k_{B}ln\left(\frac{\tau_{m}}{\tau_{0}}\right)}$$

where we denoted by *k*_B_ the Boltzmann constant, by *T*_B_ the blocking temperature of the samples, by τ_m_ the characteristic measuring time, and by τ_0_ the attempt time, while *V* is the volume of a single nanoparticle. Usually, in the experimental work, τ_m_ is hundreds of seconds for a vibrating-sample magnetometer, while τ_0_ is considered 10^−9^ s usually, so the value of $ln\left(\frac{\tau_{m}}{\tau_{0}}\right)$ can be approximated with 25 [72]. We calculated *T*_B_ values, presented in Table 3, considering that the nanoparticles can be approximated with spherical ones. The obtained values are close to room temperature for samples with La and Pr. The other studied samples have calculated blocking temperatures lower than 300 K, behavior that could be explained by non-spherical shapes of the nanoparticles.

Kurtan et al. [73] showed that the nature of intergrain exchange interactions depends on the ratio SR = *M*ᵣ/*M*ₛ, where *M*ᵣ is the remanent magnetization and *M*ₛ is the saturation magnetization. This ratio varies between 0 and 1. When R < 0.5, magnetostatic interactions dominate. At SR = 0.5, consistent with the Stoner–Wohlfarth model, the system behaves as a collection of non-interacting, randomly oriented particles. For SR > 0.5, significant exchange coupling between particles occurs. In our samples, the *M*ᵣ/*M*ₛ ratio at room temperature is below 0.5, indicating that magnetostatic interactions are the dominant mechanism.

Given the environmentally friendly sol–gel method of preparation and the magnetic properties of these compounds, our aim is to use them for technical applications with an accent on cancer diagnosis/therapy by developing magnetoplasmonic and magnetoelectric core–shell structures The aim is to locally generate heat or electric charges within the body, controlled by laser energy or/and relatively low external magnetic fields, to selectively target and affect only cancer cells. Another application to be tested is the usage of these nanoparticles in photocatalytic applications and wastewater treatment.

## 4. Conclusions

Co_0.95_R_0.05_Fe_2_O_4_ nanoparticles with R = La, Pr, Nd, Sm, and Eu were synthesized via an environmentally friendly sol–gel method. The prepared samples were studied using X-ray diffraction measurements (XRD), transmission electron microscopy (TEM), X-ray photoelectron microscopy (XPS), and magnetic measurements. All compounds were found to be single phases adopting a cubic *Fd-3m* structure. EDS analysis confirmed the presence of Co, Fe, R, and oxygen in all cases. The TEM images reveal that well-defined nanoparticles were present, with polygonal-to-spherical shapes, which tend to agglomerate, probably due to the strong magnetic interactions that outweigh the relatively weak electrostatic repulsion. TEM images show nanoparticles ranging in size from 17 nm to 25 nm, with an average diameter between 19 nm to 21 nm, closely matching the values between 18 nm and 21 nm derived from XRD measurements, suggesting that most of the nanoparticles are monodomain.

The oxidation state of Co and Fe ions in Co_0.95_R_0.05_Fe_2_O_4_ nanoparticles was investigated by XPS. The Co *2*p core-level spectra are characteristic for Co^3+^ ions, as indicated by the 2*p*_3/2_ and 2*p*_1/2_ binding energies and spin–orbit splitting values. The analysis of the Fe 2*p* core-level spectra reveals the presence of both Fe^3+^ and Fe^2+^ ions in the investigated Co_0.95_R_0.05_Fe_2_O_4_ samples. XPS valence band spectra show contributions from the 3*d* states of Fe and Co at binding energies below 3 eV, with a strong feature at about 1.6 eV from Co^3+^ ions in the octahedral sites. The density of states near the Fermi level, attributed mainly to the 3*d* states in Fe^2+^ ions, seems to be independent of the rare-earth ions used to substitute Co in Co_0.95_R_0.05_Fe_2_O_4_ nanoparticles.

The doped samples exhibit lower saturation magnetizations than the pristine sample. Very good agreement with the saturation magnetization values was obtained if we assumed that the light rare-earth ions occupy octahedral sites and their magnetic moments align parallel to those of the 3*d* transition metal ions. The ZFC-FC curves indicate that some nanoparticles remain superparamagnetic, while others exhibit ferrimagnetic ordering at room temperature, suggesting the presence of interparticle interactions. The *T*_B_ values were calculated considering that the nanoparticles can be approximated with spherical ones. The obtained values are close to room temperature for samples with La and Pr. The other studied samples have calculated blocking temperatures lower than 300 K, behavior that could be explained by non-spherical shapes of the nanoparticles.

The *M*_r_/*M*_s_ ratio at room temperature reflects the dominance of magnetostatic interactions.

## Figures and Tables

**Table 1:** Structural parameters, goodness of fit (χ^2^), and nanoparticle dimensions determined from XRD and TEM measurements of Co_0.95_R_0.05_Fe_2_O_4_ nanoparticles.

R	*a* (Å)	χ^2^	*d* (nm) XRD	*d* (nm) TEM
La	8.378(4)	1.22	20.7(1)	20.929 ± 1.573
Pr	8.375(1)	1.23	20.0(2)	20.574 ± 1.529
Nd	8.381(1)	1.22	17.9(0)	20.035 ± 1.329
Sm	8.381(0)	1.17	19.5(2)	20.567 ± 1.623
Eu	8.379(6)	1.26	19.3(7)	19.032 ± 2.046

**Table 2:** Co 2*p*_3/2_ and 2*p*_1/2_ binding energies and spin–orbit splitting Δ_SO_ determined from XPS spectra of Co_0.95_R_0.05_Fe_2_O_4_ nanoparticles.

R	2*p*_3/2_ (eV)	2*p*_1/2_ (eV)	Δ_SO_ (eV)
La	780.2 ± 0.2	795.6 ± 0.2	15.4
Pr	780.6 ± 0.2	795.9 ± 0.2	15.3
Nd	780.4 ± 0.2	795.7 ± 0.2	15.3
Sm	780.6 ± 0.2	796.0 ± 0.2	15.4
Eu	780.4 ± 0.2	795.8 ± 0.2	15.4

**Table 3:** Site occupancies of cations for Co_0.95_R_0.05_Fe_2_O_4_ samples and calculated and experimental saturation magnetization moments measured at 4.2 K and 300 K.

R	Cation Distribution							*M*_S_ (μ_B_) th T = 4.2 K	*M*_S_ (emu/g) exp. T = 4.2 K	*M*_S_ (μ_B_) exp. T = 4.2 K	*M*_S_ (emu/g) exp. T = 300 K	*M*_S_ (μ_B_) exp. T = 300 K
	Tetrahedral Sites		Octahedral Sites									
	Fe^3+^	Fe^2+^	R^3+^	Co^3+^	Co^2+^	Fe^3+^	Fe^2+^					
La	0.9	0.1	0.05	0.95	0	0.1	0.9	3	70.2597	3	63.54	2.72
Pr	0.9	0.1	0.05	0.95	0	0.1	0.9	3.16	73.4661	3.14	61.7	2.64
Nd	1	0	0.05	0.95	0	0	1	2.96	69.3367	2.97	61.3	2.62
Sm	0.78	0.22	0.05	0.94	0.01	0.23	0.77	3.28	76.6135	3.28	66.62	2.85
Eu	1	0	0.05	0.95	0	0	1	2.8	66.8424	2.87	59.02	2.53

**Table 4:** Coercive fields, remanent magnetization, the squareness ratio, *M*_r_/*M*_s_, anisotropy constant, and blocking temperature calculated using XRD and TEM data.

R	m_0_*H*_c_ (T)	*M*_r_ (emu/g)	*SR = M*_r_/*M*_s_	*K* (KJ/m^3^*)*	*T_B_* (K) XRD	*T_B_* (K) *TEM*
La	0.065	17.45	0.27	21.84823	293.95	303.82
Pr	0.0733	18.469	0.3	23.92461	290.33	316.05
Nd	0.0659	15.439	0.25	21.36985	185.91	260.69
Sm	0.0612	16.095	0.24	21.56809	242.59	284.63
Eu	0.0654	15.212	0.26	20.41891	222.67	213.52

## Data Availability

The data presented in this study are available on request from the corresponding authors.
